# Abdominal obesity and osteoarthritis risk: evaluating the association of lipid accumulation product, body roundness index, and waist triglyceride index with osteoarthritis in U.S. adults

**DOI:** 10.3389/fnut.2025.1570740

**Published:** 2025-06-19

**Authors:** Yuanchao Li, Congmin Lai, Zhiming Pan, Yanan He, Chunlong Liu

**Affiliations:** ^1^Clinical College of Acupuncture, Moxibustion, and Rehabilitation, Guangzhou University of Chinese Medicine, Guangzhou, Guangdong, China; ^2^Department of Rehabilitation Medicine, Huangpu Traditional Chinese Medicine Hospital, Guangzhou, China

**Keywords:** osteoarthritis, lipid accumulation product, body roundness index, waist triglyceride index, abdominal obesity, visceral fat

## Abstract

**Objective:**

This study explored the associations between lipid accumulation product (LAP), body roundness index (BRI), and waist triglyceride index (WTI) and osteoarthritis (OA) in U.S. adults, using data from National Health and Nutrition Examination Survey (NHANES) 2015–2018.

**Methods:**

This cross-sectional analysis included 3,611 participants aged ≥20 years. Using survey-weighted procedures, multivariable logistic regression assessed associations between anthropometric indices and OA. Smooth curve fitting evaluated non-linear relationships and threshold effects. Segmented linear regression was applied to identify potential inflection points. Subgroup analyses explored demographic and health-related variations, while receiver operating characteristic (ROC) curves assessed the discriminative capacity of these anthropometric indices.

**Results:**

Among participants, 517 (14.31%) had OA. All indices showed positive associations with OA after adjustment: LAP (OR: 1.19, CI: 1.13–1.25), BRI (OR: 1.02, CI: 1.01–1.02), and WTI (OR: 3.72, CI: 2.25–6.16). LAP significantly increased OA risk below 131.16 (OR: 1.02, *p* < 0.001) but not above. BRI demonstrated a linear relationship with OA without significant threshold effects (*p* = 0.190). WTI demonstrated dramatically increased risk above 8.72 (OR: 74.40) vs. below (OR: 4.70). Significant interactions were observed for gender with BRI (*p* = 0.0145) and hyperlipidemia with LAP (*p* = 0.0024). Stronger associations appeared in participants with lower education, non-smokers (for BRI), and those with hypertension (for WTI). BRI showed higher diagnostic accuracy [area under the curve (AUC): 0.6588].

**Conclusion:**

Central obesity-related indices demonstrate significant associations with OA prevalence in U.S. adults, with distinct threshold effects for LAP and WTI. These indices, particularly BRI, may serve as valuable screening tools for OA risk assessment in clinical practice.

## Introduction

Osteoarthritis (OA) is a prevalent degenerative joint disorder characterized by articular cartilage deterioration, osteophyte formation, and subchondral bone changes (1) that imposes substantial economic and healthcare burdens globally (2). As populations age and obesity rates rise, OA prevalence is projected to increase significantly, making the identification of modifiable risk factors crucial for public health interventions (3).

Among these risk factors, abdominal obesity has emerged as particularly significant, extending beyond mechanical loading to include complex inflammatory and metabolic pathways (4, 5). Visceral adipose tissue (VAT) functions as an active endocrine organ secreting pro-inflammatory cytokines, including tumor necrosis factor-alpha and interleukin-6 (IL-6) which promote chondrocyte apoptosis and cartilage matrix degradation (6, 7). These inflammatory processes accelerate joint degeneration and contribute to OA pathogenesis (8).

Traditional measures of obesity such as body mass index (BMI) and waist-to-hip ratio have notable limitations in accurately reflecting abdominal fat distribution and its metabolic effects (9, 10). BMI fails to account for individual fat distribution patterns, while waist circumference only partially captures abdominal adiposity, especially in the context of increasing sedentary behaviors (11). These limitations have prompted the development of novel anthropometric indices that better reflect central adiposity.

The lipid accumulation product (LAP), which combines waist circumference with triglyceride (TG) levels, has demonstrated strong predictive capability for visceral fat levels and obesity-related diseases (12). LAP provides a physiologically based metric reflecting the accumulation of lipids and their metabolic consequences, which may directly impact joint health through increased inflammatory signaling pathways. The body roundness index (BRI) offers a more comprehensive evaluation of body fat distribution by considering body width-to-height ratio (13), potentially capturing mechanical stress distribution patterns that conventional measures miss. The waist triglyceride index (WTI) offers a practical and easy composite measure that combines lipid profile with central adiposity (14).

These indices may provide more insights into OA pathogenesis than traditional measures because they better reflect the metabolically active visceral fat compartment. Elevated LAP, BRI, and WTI values potentially indicate excess visceral adiposity, which produces pro-inflammatory adipokines and cytokines (15, 16). These inflammatory mediators penetrate joint tissues, triggering cartilage degradation through increased expression of matrix metalloproteinases and promoting chondrocyte senescence and apoptosis (17). Additionally, visceral adiposity also disrupts metabolic homeostasis through insulin resistance and lipid metabolism dysregulation, leading to oxidative stress and advanced glycation end-products accumulation in joint tissues, and finally aggravates joint inflammation (18). While these indices have been associated with various health conditions including cardiovascular disease and diabetes, their relationship with OA remains underexplored (19, 20).

The National Health and Nutrition Examination Survey (NHANES) provides comprehensive health data from a representative U.S. sample, offering an opportunity to investigate these relationships (21). However, no study has comprehensively examined the associations between LAP, BRI, WTI, and OA using NHANES data.

Therefore, this study aims to explore the potential associations between these central obesity-related indices and OA among U.S. adults using NHANES 2015–2018 data. We hypothesize that elevated central obesity-related indices significantly affect OA prevalence, with potential threshold effects that could serve as clinical markers for OA risk assessment, ultimately contributing to the development of targeted prevention and treatment strategies.

## Materials and methods

### Data sources and study participants

The NHANES dataset which was utilized in this population-based cross-sectional study comprises five main components: demographic, questionnaire, laboratory, dietary, and examination data (https://www.cdc.gov/nchs/nhanes/). Data collected from the NHANES dataset (2015–2018), which is publicly available for download on the website, initially included 20,768 participants.

### Inclusion/exclusion criteria

Eligibility criteria included adults ≥20 years with complete OA diagnostic data. Exclusion criteria encompassed pregnancy, cancer diagnoses, and inflammatory arthritis (ICD-10 codes M05b-M14).

### Variables and covariates

This study considered LAP, BRI, and WTI as independent variables, with OA as the outcome of interest. Formulas of three anthropometric indexes are as follows (12–14):


(1) $$\begin{array}{l} \text{LAP}=(\text{Waist circumference }(\text{cm})\text{ - X})\\ \;\times \text{ Triglycerides }(\text{mmol/L})\;(\text{X}\\ \;=\text{ 65 for males and 58 for females}) \end{array}$$

(2) $$\begin{array}{l} \text{BRI}=364.2\;-\;365.5\;\times \\ \;\sqrt{1-{\left(\frac{(\text{Waist circumference }(\text{m})\text{/2}\pi }{0.5\;\times \text{ Height }\left(\text{m}\right)}\right)}^{2}} \end{array}$$

(3) $$\begin{array}{l} \text{WTI}=\text{ Ln }[\text{Triglycerides }(\text{mg/dl})\\ \;\times \text{ Waist circumference }(\text{cm})\text{/2}]. \end{array}$$

All formulas were applied using standardized measurements to ensure consistency and accuracy. These indicators were chosen for their capacity to represent central obesity and the pattern of body fat, particularly in the visceral abdominal region, which is closely associated with osteoarthritis.

OA was defined based on diagnosis and recorded data. Participants who met the following criteria were considered to have OA:

(1) Diagnosis as osteoarthritis made by a healthcare professional. Documentation of OA-related symptoms, including joint pain, stiffness, or swelling lasting for more than 3 months.
(2) Use of OA-specific medications or treatments. The definition used in this study aligns with standardized diagnostic criteria to ensure consistency and accuracy in identifying participants with OA (22).

When investigating the association between LAP, BRI, WTI, and OA, considering the potential influence and moderating effects of multiple covariates on these relationships is crucial. Therefore, we carefully selected 17 potential confounding variables: demographic factors (age, gender, race/ethnicity, education level, and income-to-poverty ratio), laboratory measurements [aspartate aminotransferase (AST), alanine aminotransferase (ALT), uric acid, low-density lipoprotein, high-density lipoprotein, total cholesterol (TC), blood urea nitrogen], clinical comorbidities (hypertension, diabetes, hyperlipidemia), and lifestyle behaviors (consumption, smoking status). This comprehensive adjustment approach, supported by previous epidemiological studies (2, 3, 5), helps isolate independent relationships between primary variables while minimizing confounding bias.

### Statistical analysis

R software (version 4.3.3, available at http://www.R-project.org) and EmpowerStats (version 2.0) (23). Given NHANES' complex multistage probability sampling design, appropriate survey weights were applied to all analyses to ensure nationally representative estimates. The normality of continuous variables was assessed using the Shapiro–Wilk test. Variables following normal distribution were analyzed using Student's *t*-test, while non-normally distributed variables were evaluated using the Mann–Whitney *U* test. Weighted chi-square tests were conducted to evaluate baseline characteristics across populations. To evaluate linear relationships between anthropometric indices (LAP, BRI, and WTI) and OA, multivariable logistic regression was employed as OA diagnosis is a binary outcome variable. The linearity of the relationship between continuous predictors and the logit of the outcome was examined using Box–Tidwell tests. Multicollinearity among covariates was assessed by calculating variance inflation factors (VIF), with VIF ≥ 5 indicating significant multicollinearity. When detected, redundant variables were removed from the model.

After adjusting for covariates in Model 3, Smooth curve fitting was performed to evaluate non-linear relationships between these indices and OA. Additionally, segmented linear regression models were applied to assess potential non-linear associations between OA and the three indices, identify threshold effects, and calculate inflection points. Subgroup analyses and interaction tests were conducted according to gender, race, education level, smoking, alcohol consumption, hypertension, diabetes, and hyperlipidemia to explore potential differences between different populations. Subgroup-specific interactions were tested through generalized linear models incorporating multiplicative interaction terms, with Bonferroni correction to account for multiple comparisons. Discriminative capacity was evaluated through receiver operating characteristic (ROC) curve analysis with area under the curve (AUC) comparisons to assess diagnostic performance. Statistical significance was defined as a *p*-value < 0.05. For detailed information on the adjusted models, please refer to the table notes.

## Results

### Baseline information

**Figure 1:**
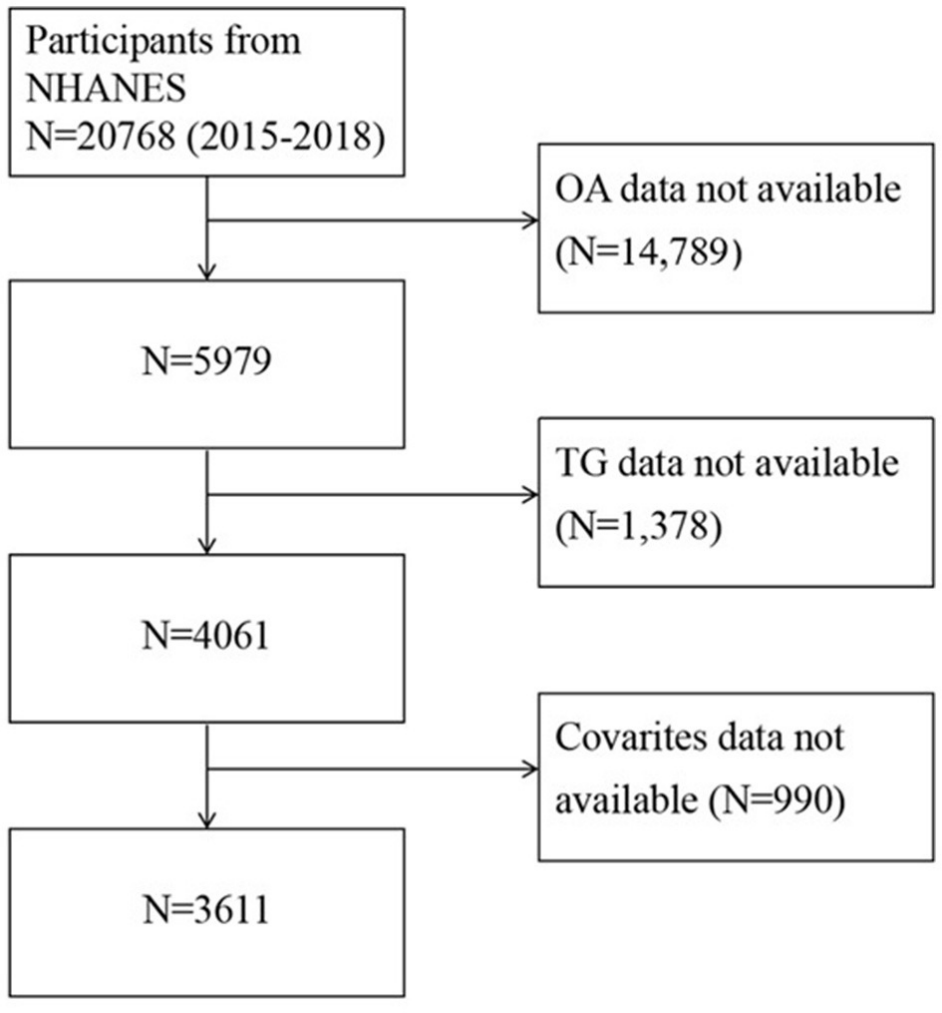
Flow chart of patient screening.

After exclusion inaccessible data (Figure 1), the final analysis included a total of 3,611 participants (1,754 men and 1,857 women). The baseline information of these participants is shown in Table 1. Among them, 172 males (33.27%) and 345 females (66.73%) were diagnosed with osteoarthritis (OA). The average age was 45.96 ± 16.88 in the non-OA group and 62.57 ± 12.82 in the OA group.

**Table 1:** Based on the baseline characteristics of the study population ascertained by NHANES from 2015 to 2018.

**Characteristics**	**Total (*N* = 3,611)**	**Non-osteoarthritis (*N* = 3,094)**	**Osteoarthritis (*N* = 517)**	***p*-value**
Age (years)	48.33 ± 17.36	45.96 ± 16.88	62.57 ± 12.82	**< 0.001**
Poverty-to-income ratio	2.51 ± 1.52	2.50 ± 1.52	2.60 ± 1.51	0.140
Body mass index (kg/m^2^)	29.22 ± 6.98	28.86 ± 6.81	31.34 ± 7.59	0.180
AST	23.55 ± 12.82	23.54 ± 12.79	23.60 ± 12.99	**< 0.001**
ALT	25.44 ± 10.69	25.49 ± 10.84	25.16 ± 9.78	0.488
Blood urea nitrogen	14.35 ± 5.58	14.01 ± 5.26	16.40 ± 6.87	**< 0.001**
Uric acid	5.43 ± 1.42	5.43 ± 1.42	5.42 ± 1.46	0.579
Total cholesterol	188.48 ± 41.23	187.61 ± 40.37	193.71 ± 45.69	0.021
Triglyceride	106.91 ± 64.50	105.51 ± 65.00	115.24 ± 60.83	**< 0.001**
Low-density lipoprotein	112.34 ± 36.06	112.29 ± 35.41	112.62 ± 39.77	**0.046**
High-density lipoprotein	54.76 ± 16.64	54.21 ± 16.07	58.04 ± 19.40	**< 0.001**
Height	166.63 ± 9.88	166.96 ± 9.87	164.66 ± 9.74	**< 0.001**
WC	99.37 ± 16.68	98.24 ± 16.32	106.10 ± 17.25	**< 0.001**
**Gender (%)**				**< 0.001**
Male	1,754 (48.57%)	1,582 (51.13%)	172 (33.27%)	
Female	1,857 (51.43%)	1,512 (48.87%)	345 (66.73%)	
**Race (%)**				**< 0.001**
Mexican American	568 (15.73%)	519 (16.77%)	49 (9.48%)	
Other Hispanic	420 (11.63%)	369 (11.93%)	51 (9.86%)	
Non-Hispanic White	1,209 (33.48%)	941 (30.41%)	268 (51.84%)	
Non-Hispanic Black	742 (20.55%)	650 (21.01%)	92 (17.79%)	
Other race	672 (18.61%)	615 (19.88%)	57 (11.03%)	
**Education level (%)**				**0.013**
< 9th Grade	313 (8.67%)	280 (9.05%)	33 (6.38%)	
9–11th Grade	419 (11.60%)	365 (11.80%)	54 (10.44%)	
High school grade	814 (22.54%)	685 (22.14%)	129 (24.95%)	
Some college or AA degree	1,095 (30.32%)	915 (29.57%)	180 (34.82%)	
College graduate or above	970 (26.86%)	849 (27.44%)	121 (23.40%)	
**Alcohol drinking (%)**				**< 0.001**
Yes	457 (12.66%)	366 (11.83%)	91 (17.60%)	
No	2,505 (69.37%)	2,147 (69.39%)	358 (69.25%)	
Other	649 (17.97%)	581 (18.78%)	68 (13.15%)	
**Hypertension (%)**				**< 0.001**
Yes	1,192 (33.01%)	877 (28.35%)	315 (60.93%)	
No	2,419 (66.99%)	2,217 (71.65%)	202 (39.07%)	
**Diabetes**				**< 0.001**
Yes	495 (13.71%)	374 (12.09%)	121 (23.40%)	
No	3,017 (83.55%)	2,648 (85.59%)	369 (71.37%)	
Other	99 (2.74%)	72 (2.33%)	27 (5.22%)	
**Smoking status (%)**				**0.001**
Yes	1,487 (41.18%)	1,221 (39.46%)	266 (51.45%)	
No	2,124 (58.82%)	1,873 (60.54%)	251 (48.55%)	
**Hyperlipidemia**				**0.009**
Yes	2,124 (58.82%)	1,793 (57.95%)	331 (64.02%)	
No	1,487 (41.17%)	1,301 (42.05%)	186 (35.98%)	
LAP	53.02 ± 42.14	51.02 ± 41.53	64.95 ± 43.85	**< 0.001**
BRI	5.57 ± 2.40	5.38 ± 2.31	6.69 ± 2.60	**< 0.001**
WHTR	0.60 ± 0.10	0.59 ± 0.10	0.65 ± 0.10	**< 0.001**
WTI	8.40 ± 0.64	8.37 ± 0.65	8.57 ± 0.58	**< 0.001**

All values are presented as proportion (%) or mean (standard error); to analyze the continuous variables, a weighted Student's *t*-test was employed, while for categorical variables, a chi-square test was utilized. Significant values are in bold. AST, aspartate aminotransferase; ALT, alanine aminotransferase; WC, waist circumference; LAP, lipid accumulation product; BRI, body roundness index; WHtR, waist-to-height ratio; WTI, waist triglyceride index.

Participants with OA displayed significantly higher levels of triglycerides (TG) and total cholesterol (TC) compared to the non-OA. Additionally, as indicated in Table 1, the OA group had significantly elevated values for all three anthropometric measures —LAP, BRI, and WTI. BMI, WC, height, and weight, were also higher in the OA group. The prevalence of smoking, alcohol consumption, hypertension, hyperlipidemia and diabetes were notably higher in OA participants.

### Associations between three anthropometric indexes and OA

Table 2 presents result after adjustments for potential confounding factors. It is apparent that with significant increases in OA risk associated with higher LAP, BRI, and WTI (*p* < 0.0001): For LAP the ORs were 1.01 (CI: 1.00–1.01) in model 1, 1.01 (CI: 1.00–1.01) in model 2 and 1.02 (CI: 1.01–1.02) in model 3. For BRI, the ORs were 1.22 (CI: 1.18–1.27) in model 1, 1.18 (1.13, 1.23) in model 2 and 1.19 (1.13, 1.25) in model 3. For WTI, the ORs were 1.64 (CI: 1.41–1.90) in model 1, 1.53 (CI: 1.28–1.83) in model 2 and 3.72 (CI: 2.25–6.16) in model 3.

**Table 2:** Multivariable logistic regression models for the association between LAP, BRI, and WTI and osteoarthritis in adults in the NHANES 2015–2018.

**Exposure**	**Crude model (model 1) OR (95% CI) *p*-value**	**Partially adjusted model (model 2) OR (95% CI) *p*-value**	**Fully adjusted model (model 3) OR (95% CI) *p*-value**
LAP	1.01 (1.00, 1.01) < 0.0001	1.01 (1.00, 1.01) < 0.0001	1.02 (1.01, 1.02) < 0.0001
BRI	1.22 (1.18, 1.27) < 0.0001	1.18 (1.13, 1.23) < 0.0001	1.19 (1.13, 1.25) < 0.0001
WTI	1.64 (1.41, 1.90) < 0.0001	1.53 (1.28, 1.83) < 0.0001	3.72 (2.25, 6.16) < 0.0001

Model 1, no covariates were adjusted.

Model 2, age, gender, and race were adjusted.

Model 3, age, gender, race, education level, income-to-poverty ratio, hypertension, AST, ALT, uric acid, diabetes, alcohol drinking, smoking status, low-density lipoprotein, high-density lipoprotein, blood urea nitrogen, total cholesterol, and hyperlipidemia were adjusted.

OR, odds ratio; 95% CI, 95% confidence interval.

### Analysis of non-linearity and threshold effects between LAP, BRI, WTI, and osteoarthritis

A significant threshold effect for LAP was identified at 131.16 (Table 3, log-likelihood ratio test, *p* < 0.001). Below this value, LAP was positively associated with the likelihood of osteoarthritis [1.02 (CI: 1.02–1.03), *p* < 0.001], while no significant relationship was found above this threshold [1.00 (CI: 0.99–1.01), *p* = 0.826].

**Table 3:** Threshold effect analysis of LAP, BRI, and WTI and osteoarthritis using a two-piecewise logistic regression model in adults in the NHANES 2015–2018.

**Threshold effect analysis**	**Cataract OR (95%CI) *p*-value**
**LAP**	
Fitting by the standard linear model	1.02 (1.01, 1.02) < 0.001
Inflection point of LAP (K)	131.16
< K slope	1.02 (1.02, 1.03) < 0.001
>K slope	1.00 (0.99, 1.01) 0.826
Log-likelihood ratio test	< 0.001
**BRI**	
Fitting by the standard linear model	1.19 (1.14, 1.25) < 0.0001
Inflection point of VAI (K)	2.88
< K slope	0.63 (0.26, 1.54) 0.315
>K slope	1.20 (1.14, 1.26) < 0.001
Log-likelihood ratio test	0.191
**WTI**	
Fitting by the standard linear model	3.63 (2.20, 5.99) < 0.001
Inflection point of AIP (K)	8.72
< K slope	4.70 (2.84, 7.78) < 0.001
>K slope	74.40 (22.91, 241.60) < 0.001
Log-likelihood ratio test	< 0.001

Age, gender, race, education level, income-to-poverty ratio, hypertension, AST, ALT, uric acid, diabetes, alcohol drinking, smoking status, low-density lipoprotein, high-density lipoprotein, blood urea nitrogen, total cholesterol, and hyperlipidemia were adjusted.

Non-linear relationships, including threshold and saturation effects, were explored using generalized additive models (GAMs).

95% CI, 95% confidence interval; OR, odds ratio.

For BRI, the standard linear model showed a significant positive association with osteoarthritis risk [1.19 (CI: 1.14–1.25), *p* < 0.0001]. The threshold effect was not statistically significant (log-likelihood ratio test, *p* = 0.191).

**Figure 2:**
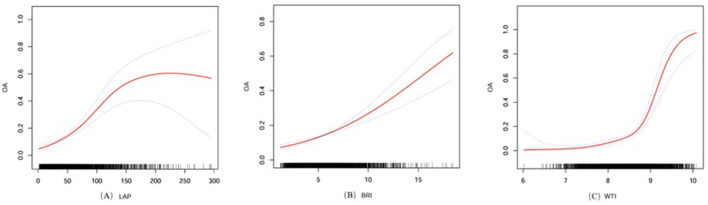
The nonlinear associations between the LAP **(A)**, BRI **(B)**, WTI **(C)** and OA. The solid red line represents the smooth curve fit between variables. Blue bands represent the 95% confidence interval from the fit. Age, gender, race, education level, income-to-poverty ratio, hypertension, AST, ALT, uric acid, diabetes, alcohol drinking, smoking status, low-density lipoprotein, high-density lipoprotein, blood urea nitrogen, total cholesterol, and hyperlipidemia were adjusted.

For WTI, a significant threshold effect was confirmed at 8.72 (log-likelihood ratio test, *p* < 0.001). Below this threshold, a strong positive correlation with osteoarthritis was observed [4.70 (CI: 2.84–7.78), *p* < 0.001], with the association becoming dramatically stronger above the threshold [74.40 (CI: 22.91–241.60), *p* < 0.001] (Figure 2).

### Subgroup analysis of LAP, BRI, WTI, and osteoarthritis prevalence

Significant positive associations between LAP, BRI, WTI, and the prevalence of osteoarthritis were consistently observed across subgroups categorized by gender, race, education, smoking status, alcohol intake, hypertension, and diabetes (*p* < 0.01 for most subgroups) (Supplementary Table S1). Significant interactions were identified for gender with BRI (*p* interaction = 0.0145), race with BRI (*p* interaction = 0.0394), smoking status with BRI (*p* interaction = 0.0348), hypertension with WTI (*p* interaction = 0.0438), and hyperlipidemia with LAP (*p* interaction = 0.0024).

The associations were significant in both males and females, with males showing slightly stronger associations for BRI (OR 1.29, 95% CI: 1.19–1.41) and WTI (OR 4.80, 95% CI: 2.66–8.66) compared to females (BRI: OR 1.15, 95% CI: 1.09–1.22; WTI: OR 3.29, 95% CI: 1.98–5.46). Across racial categories, Mexican Americans showed the strongest association with WTI (OR 6.40, 95% CI: 2.30–17.50), while Non-Hispanic Black people had the strongest association with BRI (OR 1.31, 95% CI: 1.18–1.46).

Participants with lower education levels (< 9th grade) showed notable associations with LAP (OR 1.03, 95% CI: 1.01–1.04) and WTI (OR 6.40, 95% CI: 1.70–23.90). For smoking status, non-smokers demonstrated stronger associations with BRI (OR 1.24, 95% CI: 1.17–1.32) compared to smokers (OR 1.13, 95% CI: 1.06–1.21).

The association between WTI and OA was significantly stronger in individuals with hypertension (OR 4.90, 95% CI: 2.70–8.60) compared to those without (OR 3.30, 95% CI: 1.90–5.40). For hyperlipidemia, a significant interaction was observed with LAP (*p* interaction = 0.0024), with stronger associations in participants without hyperlipidemia (OR 1.028, 95% CI: 1.019–1.031) compared to those with the condition (OR 1.016, 95% CI: 1.010–1.021).

ROC curve analysis was performed to evaluate the diagnostic utility of the adiposity indices for OA. The BRI showed an area under the curve (AUC) of 0.6588 (95% CI: 0.6341–0.6835, *p* < 0.001), the LAP demonstrated an AUC of 0.6098 (95% CI: 0.5844–0.6351, *p* < 0.001), and the WTI had an AUC of 0.5898 (95% CI: 0.5643–0.6152, *p* < 0.001). Additional performance metrics are presented in Table 4.

**Table 4:** ROC curves of LAP, BRI and WTI index for OA.

**Variable**	**Sensitivity**	**Specificity**	**ROC area (AUC)**	**95%CI low**	**95%CI up**	***p*-value**
LAP	0.4642	0.7001	0.6098	0.5844	0.6351	< 0.001
BRI	0.5880	0.6464	0.6588	0.6341	0.6835	< 0.001
WTI	0.3946	0.7346	0.5898	0.5643	0.6152	0.050

AUC, area under curve; ROC, receiver operating characteristics curve; 95% CI, 95% confidence interval.

## Discussion

Obesity has long been established as a primary modifiable risk factor for OA development and progression (3). Traditional obesity metrics like body mass index (BMI) have demonstrated consistent associations with OA risk across populations (10). However, these conventional indicators have significant limitations in differentiating between adipose tissue distribution patterns, particularly failing to accurately quantify visceral adiposity (9), which may play a crucial role in OA pathogenesis beyond simple mechanical loading effects.

Increasing evidence suggests that visceral adiposity contributes to OA through multiple pathophysiological mechanisms. Visceral adipose tissue functions as an active endocrine organ, secreting pro-inflammatory adipokines and cytokines that promote systemic low-grade inflammation (6, 7). This inflammatory state can directly impact articular cartilage metabolism, synovial inflammation, and subchondral bone remodeling, accelerating joint degeneration independent of mechanical factors (8). Recent studies have demonstrated that even individuals with normal BMI but metabolic syndrome (MetS) exhibit significantly higher OA prevalence and progression rates compared to metabolically healthy counterparts, highlighting the strong connection between lipid metabolism dysregulation and OA pathogenesis regardless of overall weight status (24).

While the link between obesity and OA is well-established, recent advances in research methodologies have provided deeper insights into the specific metabolic mechanisms involved. Metabolomics research has identified distinct lipid profiles in OA patients that correlate with disease severity, indicating that lipid metabolism abnormalities may contribute to OA pathogenesis (25). Interestingly, these metabolic disturbances appear to precede radiographic OA changes, suggesting they may serve as early biomarkers for OA risk assessment (26). Additionally, epidemiological studies have consistently reported that central obesity metrics, including waist circumference and waist-to-hip ratio, demonstrate stronger associations with OA risk than BMI alone, particularly for knee OA (27). These observations highlight the importance of comprehensively evaluating both lipid metabolism parameters and abdominal fat distribution patterns when assessing OA risk, suggesting that indices incorporating both elements may offer superior predictive value.

Based on these findings, researchers have developed more sophisticated metrics that better capture both metabolic dysfunction and fat distribution patterns relevant to OA pathogenesis. LAP, BRI, and WTI—offer potentially superior methods for quantifying metabolically active visceral adiposity compared to traditional anthropometric measures (12–14). Each index incorporates different components of body fat distribution and metabolic parameters, potentially capturing unique aspects of adiposity-related OA risk. Previous investigations have demonstrated strong correlations between these indices and various cardiometabolic conditions including diabetes, hypertension, cardiovascular disease, and even acute pancreatitis, many of which share inflammatory pathways with OA (19, 28). However, comprehensive research examining these specific indices in relation to OA risk remains limited.

Given these established connections between visceral adiposity, metabolic dysfunction, and OA, we hypothesized that LAP, BRI, and WTI would demonstrate significant associations with OA risk in a nationally representative sample. This cross-sectional study aims to investigate these relationships while identifying potential threshold effects and subgroup variations that might inform clinical risk assessment strategies. Our findings demonstrate significant associations between three readily accessible abdominal fat distribution indices—LAP, BRI, and WTI—and osteoarthritis risk in U.S. adults. Participants with OA exhibited markedly higher values for all three indices compared to non-OA counterparts, even after adjusting for covariates which is consistent with the results reported by previous studies (29, 30). Importantly, LAP showed a positive association with OA risk up to 131.16, beyond which the relationship became non-significant; BRI demonstrated no significant association below 2.88, but a strong positive correlation above this threshold; and WTI revealed a dramatic increase in association strength after exceeding 8.72, with the odds ratio jumping from 4.70 below the threshold to 74.40 above it. Subgroup analyses confirmed robust associations across demographic and clinical characteristics, with specific interactions observed for gender with BRI and hyperlipidemia with LAP. Stronger associations were observed in participants with lower education levels, non-smokers (for BRI), individuals with hypertension (particularly for WTI), and participants without hyperlipidemia (for LAP).

Among the three indices examined, each demonstrated distinct patterns of association with OA that warrant detailed examination. LAP, a composite indicator combining waist circumference and triglyceride levels, represents a powerful marker for assessing central lipid accumulation and metabolic dysfunction. Recent research from NHANES 2017–2020 revealed an inverse U-shaped association between LAP levels and OA prevalence, with LAP functioning as an independent risk factor for OA when below 120.00 cm × mmol/L (31). Our findings further establish its relationship with OA, particularly below the threshold value of 131.16, beyond which the association plateaus. This threshold phenomenon likely reflects the biological concept of “adiposity threshold” where adipose tissue expansion exceeds its vascular supply, triggering hypoxia-induced adipocyte dysfunction (32). The resulting cellular stress activates the unfolded protein response and stimulates NOD-, LRR- and pyrin domain-containing protein 3 (NLRP3) inflammasome activation, perpetuating synovial inflammation through pro-IL-1β processing and adipokine dysregulation (e.g., leptin resistance, adiponectin suppression) (33). Mechanistically, elevated LAP values are associated with visceral adiposity functioning as an active endocrine organ (15), secreting pro-inflammatory cytokines that contribute to systemic low-grade inflammation. Chondrocytes exposed to high triglyceride concentrations exhibit increased oxidative stress markers and reduced expression of cartilage matrix components (17), directly impacting cartilage homeostasis (34). Additionally, lipid toxicity from excessive triglycerides can impair mitochondrial function in joint tissues (6), accelerating cellular senescence and apoptosis of chondrocytes through ceramide accumulation and endoplasmic reticulum stress (35).

While LAP captures important aspects of lipid accumulation, BRI offers complementary insights by focusing on anatomical dimensions of adiposity. BRI combines height and central body fat percentage, reflecting anatomical stress on joints which is relevant to structural damage in OA (13). The biomechanical significance of BRI lies in its ability to capture alterations in body mass distribution that affect joint loading patterns (36), with odds ratios as high as 2.235 (95% CI: 1.796–2.781) for identifying metabolic syndrome risk (37). The elliptical nature of the BRI calculation specifically accounts for how abdominal fat shifts the center of gravity anteriorly, increasing anterior-posterior shear forces on weight-bearing joints, particularly knees and hips (38). Beyond purely mechanical effects, these altered loading patterns stimulate chondrocyte mechanoreceptors, particularly ion channels such as transient receptor potential vanilloid 4 (TRPV4) and Piezo 1/2, which respond to abnormal pressure by initiating inflammatory signaling pathways that further contribute to matrix degradation (39). Furthermore, central obesity, which BRI specifically targets, induces a chronic inflammatory state characterized by abnormal adipokine profiles, including leptin, adiponectin, and resistance (40). These adipokines have been consistently detected at elevated levels in the synovial fluid of OA patients and directly influence cartilage metabolism. Our study provides additional support for BRI as a valuable tool in OA risk stratification.

Beyond the mechanical factors captured by BRI, metabolic aspects of adiposity are further illuminated by examining WTI, which offers yet another perspective on OA risk. WTI, combining waist circumference and triglycerides through logarithmic transformation and less prone to confounding by lean mass (41), may offer complementary information to both LAP and BRI. Our study revealed a notable threshold effect where WTI values exceeding 8.72 were associated with a dramatic increase in OA risk, with the odds ratio jumping from 4.70 below the threshold to 74.40. above it. This non-linear relationship might correspond with a metabolic switch from subcutaneous to ectopic fat deposition, including muscle, and synovial tissues (42). Such ectopic deposition in joint-adjacent tissues alters the local lipid profile of synovial fluid, disrupting lubrication properties and increasing friction coefficients within the joint (43). The logarithmic transformation applied in WTI calculation may capture these non-linear relationships between visceral adiposity, lipid metabolism, and inflammatory pathways, potentially amplifying its association with OA compared to other indices. WTI can easily identify individuals who have significant visceral fat accumulation and metabolic disruptions which are key contributors to joint degeneration, even when they may not exhibit high overall body mass (41).

The differential associations observed across these three indices highlight the multifaceted nature of adiposity's contribution to OA risk. Meta-analyses comparing various adiposity indicators have demonstrated that indices incorporating both anthropometric measurements and lipid profiles often provide superior diagnostic accuracy compared to traditional measures such as BMI, waist circumference, or waist-to-height ratio alone (44). LAP, BRI, and WTI each capture distinct aspects of metabolic health—LAP excels at assessing visceral lipid accumulation, BRI provides superior body shape assessment, and WTI offers enhanced sensitivity to triglyceride-related metabolic dysfunction (45). By examining all three indices concurrently, clinicians can more comprehensively evaluate metabolic profiles and accurately identify patients at elevated OA risk. From a clinical perspective, the identified thresholds for LAP (131.16) and WTI (8.72) serve as valuable screening tools in primary care and rheumatology settings. These thresholds can inform risk stratification protocols, helping determine which patients might benefit most from preventive interventions targeting joint protection and weight management (21). Using routine laboratory tests and simple anthropometric measurements, these indices are easily implemented in clinical practice, supporting our understanding of OA as a multifactorial condition influenced by diverse metabolic and inflammatory pathways.

Our subgroup analyses revealed remarkable consistency in the associations between adiposity indices and OA across diverse demographic and clinical subpopulations, reinforcing the generalizability of our findings. Notably, gender-specific interactions with BRI suggest potential hormonal or body composition influences on adiposity's relationship with OA risk (46). The stronger associations observed in participants with lower education levels highlight important socioeconomic dimensions in OA risk profiling, while the significant interaction between hyperlipidemia and LAP provides additional insights. This finding suggests that in patients with pre-existing dyslipidemia, additional fat accumulation (as measured by LAP) may have a smaller contribution to OA risk, possibly because hyperlipidemia has already activated inflammatory and metabolic pathways that promote OA development (12, 32). In contrast, in metabolically healthy individuals, an increase in LAP values may represent a more significant transition from normal to pathological states, thus showing a stronger association with OA risk.

Regarding discriminative capacity, ROC analysis showed BRI had the highest association strength, followed by LAP and WTI. BRI's higher AUC in this cross-sectional analysis may reflect its ability to better capture central adiposity distribution patterns that are associated with joint loading and inflammatory processes relevant to OA. To address collinearity, we calculated variance inflation factors (VIF ≥ 5) and removed problematic variables, while our sequential adjustment across multiple models assessed estimate stability. This rigorous approach to multicollinearity assessment is widely accepted in epidemiological research and provides sufficient protection against inflated standard errors and unstable coefficient estimates (23).

This study has several strengths. First, we utilized data from NHANES, a nationally representative survey with standardized protocols for data collection, enhancing the generalizability of our findings. Second, we comprehensively analyzed three indices (LAP, BRI, and WTI) that reflect different aspects of abdominal adiposity and lipid metabolism in relation to OA, providing multifaceted evidence on the associations between metabolic and anatomical components of obesity and OA prevalence. Third, we employed sophisticated statistical methods to explore both linear and non-linear relationships, identify threshold effects, and assess diagnostic performance through ROC analysis and extensive subgroup evaluations. However, there are some limitations. First, the cross-sectional design prevents establishing causality between these adiposity indices and OA. We cannot determine whether abnormal LAP, BRI, and WTI values precede OA development or result from post-diagnosis lifestyle changes. Second, self-reported OA diagnosis may introduce recall bias (47). Third, while we addressed multicollinearity using variance inflation factors, our analysis was limited to conventional regression models. Future studies comparing multiple regression approaches could further validate the robustness of these associations. Finally, we lacked information on OA severity and joint-specific involvement, which may have provided additional insights.

## Conclusions

This cross-sectional study elucidates the significant associations between three abdominal fat distribution indices—LAP, BRI, and WTI—and osteoarthritis (OA) risk in U.S. adults. Participants with OA exhibited markedly higher values for all three indices compared to non-OA counterparts, even after adjusting for covariates. Non-linear threshold effects were identified for LAP and WTI: LAP demonstrated a saturation point (131.16) beyond which the association with OA risk became non-significant, while WTI showed a threshold (8.72) above which OA risk escalated exponentially (from OR 4.70 to 74.40). In contrast, BRI showed a linear relationship with OA risk, with no significant threshold effect (log-likelihood ratio test, *p* = 0.190). Subgroup analyses confirmed robust associations across diverse demographic and clinical characteristics, with specific interactions observed for gender with BRI and hyperlipidemia with LAP. Stronger associations were noted in participants with lower education levels, non-smokers (for BRI), individuals with hypertension (particularly for WTI), and participants without hyperlipidemia (for LAP). The identified thresholds for LAP and WTI may serve as practical screening tools for identifying individuals at higher OA risk and as targets for risk stratification in clinical settings. Since these indices can be easily calculated from routine laboratory tests and anthropometric measurements, they offer a potentially cost-effective approach to risk assessment. In future research, it would be valuable to explore whether different management approaches, such as dietary modifications for individuals with elevated LAP values or specific exercise programs for those with high WTI values. While our cross-sectional design limits causal inference, these results generate important hypotheses for future longitudinal and interventional studies examining.

## Data Availability

The datasets presented in this study can be found in online repositories. The names of the repository/repositories and accession number(s) can be found at: https://www.cdc.gov/nchs/nhanes/.
