# Supplementary material for: Abdominal obesity and osteoarthritis risk: evaluating the association of lipid accumulation product, body roundness index, and waist triglyceride index with osteoarthritis in U.S. adults
Source: Front Nutr. 2025 Jun 19;12:1570740. doi: 10.3389/fnut.2025.1570740 (PMC12221944; doi:10.3389/fnut.2025.1570740)
Supplement: Supplementary file 1 [file Table_1.docx]

### Table S1

Stratified analysis of the correlation between LAP, BRI, and WTI and Osteoarthritis in adults in the NHANES 2015–2018

| **Subgroup** | **OR(95%CI)**  **,*P*-value** | ***P* interaction** | **OR(95%CI)**  **,*P*-value** | ***P* interaction** | **OR(95%CI)**  **,*P*-value** | ***P* interaction** |
| --- | --- | --- | --- | --- | --- | --- |
|  | **LAP** |  | **BRI** |  | **WTI** |  |
| **Gender** |  | 0.307 |  | 0.0145 |  | 0.0562 |
| Male | 1.02 (1.01, 1.02) <0.0001 |  | 1.29 (1.19, 1.41) <0.0001 |  | 4.80 (2.66, 8.66) <0.0001 |  |
| Female | 1.02 (1.01, 1.02) <0.0001 |  | 1.15 (1.09, 1.22) <0.0001 |  | 3.29 (1.98, 5.46) <0.0001 |  |
| **Race** |  | 0.8250 |  | 0.0394 |  | 0.1069 |
| Mexican American | 1.01 (1.00, 1.03) 0.0074 |  | 1.05 (0.90, 1.23) 0.4759 |  | 6.40 (2.30, 17.50) 0.0003 |  |
| Other Hispanic | 1.02 (1.01, 1.03) 0.0002 |  | 1.31 (1.11, 1.54) 0.0010 |  | 4.80 (1.80, 12.50) 0.0015 |  |
| Non-Hispanic White | 1.02 (1.01, 1.02) <0.0001 |  | 1.13 (1.05, 1.22) 0.0026 |  | 5.10 (2.70, 9.50) <0.0001 |  |
| Non-Hispanic Black | 1.02 (1.01, 1.03) 0.0004 |  | 1.31 (1.18, 1.46) <0.0001 |  | 2.97 (1.30, 5.00) 0.0047 |  |
| Other Race | 1.02 (1.01, 1.03) 0.0056 |  | 1.26 (1.08, 1.50) 0.0028 |  | 2.29 (0.98, 5.32) 0.0604 |  |
| **Education Level** |  | 0.0552 |  | 0.3629 |  | 0.3921 |
| Less Than 9th Grade | 1.03 (1.01, 1.04) 0.0001 |  | 1.30 (1.00, 1.60) 0.0451 |  | 6.40 (1.70, 23.90) 0.0055 |  |
| 9-11th Grade | 1.02 (1.01, 1.03) 0.0011 |  | 1.30(1.10, 150) 0.0033 |  | 2.50 (0.90, 6.60) 0.0661 |  |
| High School Grad | 1.02 (1.01, 1.03) 0.0043 |  | 1.20 (1.10, 1.30) 0.0033 |  | 4.10 (2.00, 8.20) <0.0001 |  |
| Some College or AA degree | 1.01 (1.01, 1.02) 0.0003 |  | 1.13 (1.004, 1.20) 0.0044 |  | 2.90 (1.60, 5.40) 0.0007 |  |
| College Graduate or above | 1.02 (1.01, 1.03) <0.0001 |  | 1.30 (1.20, 1.40) <0.0001 |  | 4.70 (2.50, 9.0) <0.0001 |  |
| **Smoke** |  | 0.1309 |  | 0.0348 |  | 0.3019 |
| YES | 1.02 (1.01, 1.02) <0.0001 |  | 1.13 (1.06, 1.21) 0.0004 |  | 3.30 (2.00, 5.70) <0.0001 |  |
| NO | 1.02 (1.01, 1.03) <0.0001 |  | 1.24 (1.17, 1.32) <0.0001 |  | 4.10 (2.40, 7.00) <0.0001 |  |
| **Alcohol drinking** |  | 0.3086 |  | 0.9156 |  | 0.1013 |
| YES | 1.01 (1.00, 1.02) 0.0027 |  | 1.20 (1.02, 1.31) 0.0209 |  | 2.00 (1.00, 4.20) 0.0566 |  |
| NO | 1.02 (1.01, 1.02) <0.0001 |  | 1.20 (1.12, 1.26) <0.0001 |  | 4.20 (2.40, 7.10) <0.0001 |  |
| Other | 1.02 (1.01, 1.03) 0.0002 |  | 1.20 (1.04, 1.36) 0.0135 |  | 3.60 (1.50, 8.40) 0.0035 |  |
| **Hypertension** |  | 0.2984 |  | 0.5471 |  | 0.0438 |
| YES | 1.02 (1.01, 1.02) <0.0001 |  | 1.21 (1.13, 1.29) <0.0001 |  | 4.90 (2.70, 8.60) <0.0001 |  |
| NO | 1.02 (1.01, 1.02) <0.0001 |  | 1.18 (1.10, 1.26) <0.0001 |  | 3.30(1.90, 5.40) <0.0001 |  |
| **Diabetes** |  | 0.7343 |  | 0.4950 |  | 0.9023 |
| YES | 1.02 (1.01, 1.02) <0.0001 |  | 1.30 (1.10, 1.40) <0.0001 |  | 3.30 (150, 6.90) 0.0020 |  |
| NO | 1.02 (1.01, 1.02) <0.0001 |  | 1.20 (1.11, 1.30) <0.0001 |  | 3.80 (2.20, 6.50) <0.0001 |  |
| Other | 1.02 (1.00, 1.04) 0.0949 |  | 1.20 (0.90, 1.52) 0.3114 |  | 3.70 (0.7, 18.50) 0.1128 |  |
| **Hyperlipidemia** |  | 0.0024 |  | 0.1928 |  | 0.7647 |
| YES | 1.01 (1.01, 1.02) <0.0001 |  | 1.16 (1.09, 1.23) <0.0001 |  | 3.95 (2.07, 7.52) <0.0001 |  |
| NO | 1.02 (1.01, 1.03) <0.0001 |  | 1.23 (1.14, 1.34) <0.0001 |  | 3.65 (2.17, 6.12) <0.0001 |  |

The results show that the subgroup analysis was adjusted for all presented covariates except the effect modifier.

Stratified analysis was analyzed using generalized linear models (GLMs) with interaction terms.

Abbreviations: 95% CI, 95% confidence interval; OR, odds ratio.
